# Supplementary material for: The engaging nature of interactive gestures
Source: PLoS One. 2020 Apr 23;15(4):e0232128. doi: 10.1371/journal.pone.0232128 (PMC7179864; doi:10.1371/journal.pone.0232128)
Supplement: S1 Table — (DOCX) [file pone.0232128.s001.docx]

**Supplementary Table S1**. Average Response Times and Accuracy per stimulus at the Go/No-Go Task.

| **Stimulus number** | **Gesture-type Int/Comm** | **Model's Gender**  **(Male/Female)** | **Model's hand (Left/Right)** | **Gesture** | **Mean Accuracy** | **SD Accuracy** | **Mean Reaction Times** | **SD Reaction Times** |
| --- | --- | --- | --- | --- | --- | --- | --- | --- |
| 1 | int | Male | Right | handshake | 0.83 | 0.24 | 623.04 | 126.55 |
| 2 | int | Male | Right | highfive | 0.86 | 0.18 | 660.54 | 98.55 |
| 3 | int | Male | Right | request | 0.88 | 0.24 | 635.57 | 96.09 |
| 4 | int | Female | Right | handshake | 0.92 | 0.14 | 643.48 | 100.51 |
| 5 | int | Female | Right | highfive | 0.89 | 0.19 | 665.43 | 94.11 |
| 6 | int | Female | Right | request | 0.92 | 0.13 | 643.87 | 100.81 |
| 7 | com | Male | Right | Ok | 0.95 | 0.08 | 639.01 | 98.77 |
| 8 | com | Male | Right | peace | 0.90 | 0.16 | 632.57 | 126.97 |
| 9 | com | Male | Right | thumbsup | 0.87 | 0.21 | 635.79 | 121.87 |
| 10 | com | Female | Right | Ok | 0.82 | 0.20 | 651.75 | 80.49 |
| 11 | com | Female | Right | peace | 0.96 | 0.07 | 629.89 | 107.86 |
| 12 | com | Female | Right | thumbsup | 0.95 | 0.08 | 648.50 | 94.82 |
