# Supplementary material for: The engaging nature of interactive gestures
Source: PLoS One. 2020 Apr 23;15(4):e0232128. doi: 10.1371/journal.pone.0232128 (PMC7179864; doi:10.1371/journal.pone.0232128)
Supplement: S2 Table — (DOCX) [file pone.0232128.s002.docx]

**Supplementary Table S2**. Model selection for the analysis of Accuracy and Reaction Times in each Experiment.

| **(a) EXPERIMENT 1** | | | | |
| --- | --- | --- | --- | --- |
| **Accuracy** | | | | |
| **Model** | **df** | **AIC** | **χ^2^** | ***p*** |
| ACC ~ 1 + (1 \| Sogg) + (1 \| Stimulus) | 3 | 1842.1 |  |  |
| **ACC ~ 1 + (1 \| Sogg) + (1 \| Stimulus) + Compatibility** | **4** | **1803.1** | **40.93** | **< . 001*** |
| ACC ~ 1 + (1 \| Sogg) + (1 \| Stimulus) + Compatibility * Gesture-type | 6 | 1806.5 | 0.66 | 0.7190 |
| ACC ~ 1 + (1 \| Sogg) + (1 \| Stimulus) + Compatibility * Gesture-type * ResponseSide | 10 | 1810.2 | 4.27 | 0.3707 |
| **Response Times** | | | | |
| **Model** | **df** | **AIC** | **χ^2^** | ***p*** |
| RT ~ 1 + (1 \| Sogg) + (1 \| Stimulus) | 4 | -7690.0 |  |  |
| RT ~ 1 + (1 \| Sogg) + (1 \| Stimulus) + Compatibility | 5 | -7732.7 | 44.71 | < .001 |
| RT ~ 1 + (1 \| Sogg) + (1 \| Stimulus) + Compatibility * Gesture-type | 7 | -7733.3 | 4.64 | 0.0985 |
| **RT ~ 1 + (1 \| Sogg) + (1 \| Stimulus) + Compatibility * Gesture-type * ResponseSide** | **11** | **-7745.3** | **20.00** | **< .001*** |
| **(b) EXPERIMENT 2** | | | | |
| **Accuracy** | | | | |
| **Model** | **df** | **AIC** | **χ^2^** | ***p*** |
| ACC ~ 1 + (1 \| Sogg) + (1 \| Stimulus) | 3 | 1826.9 |  |  |
| **ACC ~ 1 + (1 \| Sogg) + (1 \| Stimulus) + Compatibility** | **4** | **1748.4** | **80.45** | **< . 001*** |
| ACC ~ 1 + (1 \| Sogg) + (1 \| Stimulus) + Compatibility * Gesture-type | 6 | 1752.1 | 0.37 | 0.8314 |
| ACC ~ 1 + (1 \| Sogg) + (1 \| Stimulus) + Compatibility * Gesture-type * PictureHemifield | 10 | 1756.7 | 3.34 | 0.5030 |
| **Response Times** | | | | |
| **Model** | **df** | **AIC** | **χ^2^** | ***p*** |
| RT ~ 1 + (1 \| Sogg) + (1 \| Stimulus) | 4 | -9713.8 |  |  |
| RT ~ 1 + (1 \| Sogg) + (1 \| Stimulus) + Compatibility | 5 | -9822.7 | 110.89 | < .001* |
| **RT ~ 1 + (1 \| Sogg) + (1 \| Stimulus) + Compatibility * Gesture-type** | **7** | **-9828.2** | **9.52** | **0.008*** |
| RT ~ 1 + (1 \| Sogg) + (1 \| Stimulus) + Compatibility * Gesture-type * PictureHemifield | 11 | -9827.5 | 7.28 | 0.1220 |
| **(c) EXPERIMENT 3** | | | | |
| **Accuracy** | | | | |
| **Model** | **df** | **AIC** | **χ^2^** | ***p*** |
| ACC ~ 1 + (1 \| Sogg) + (1 \| Stimulus) | 3 | 1351.3 |  |  |
| **ACC ~ 1 + (1 \| Sogg) + (1 \| Stimulus) + Compatibility** | **4** | **1320.7** | **32.66** | **< .001*** |
| ACC ~ 1 + (1 \| Sogg) + (1 \| Stimulus) + Compatibility * Gesture-type | 6 | 1321.3 | 3.39 | 0.1835 |
| ACC ~ 1 + (1 \| Sogg) + (1 \| Stimulus) + Compatibility * Gesture-type * PictureHemifield | 10 | 1326.3 | 2.94 | 0.5686 |
| **Response Times** | | | | |
| **Model** | **df** | **AIC** | **χ^2^** | ***p*** |
| RT ~ 1 + (1 \| Sogg) + (1 \| Stimulus) | 4 | -5215.0 |  |  |
| RT ~ 1 + (1 \| Sogg) + (1 \| Stimulus) + Compatibility | 5 | -5278.3 | 65.31 | < .001* |
| **RT ~ 1 + (1 \| Sogg) + (1 \| Stimulus) + Compatibility * Gesture-type** | **7** | **-5288.1** | **13.76** | **0.001*** |
| RT ~ 1 + (1 \| Sogg) + (1 \| Stimulus) + Compatibility * Gesture-type * PictureHemifield | 11 | -5282.8 | 2.68 | 0.6127 |
