# Supplementary material for: The engaging nature of interactive gestures
Source: PLoS One. 2020 Apr 23;15(4):e0232128. doi: 10.1371/journal.pone.0232128 (PMC7179864; doi:10.1371/journal.pone.0232128)
Supplement: S3 Table — (DOCX) [file pone.0232128.s003.docx]

**Supplementary Table S3.** The Stimulus-type variable that was used as random intercept to account for the between-stimuli variability.

| **Stimulus number** | **Gesture-type (Interactive/Communicative)** | **Model's Gender**  **(Male/Female)** | **Model's hand (Left/Right)** | **Gesture** |
| --- | --- | --- | --- | --- |
| 1 | int | Male | Right | handshake |
| 2 | int | Male | Right | highfive |
| 3 | int | Male | Right | request |
| 1 | int | Male | Left | handshake |
| 2 | int | Male | Left | highfive |
| 3 | int | Male | Left | request |
| 4 | int | Female | Right | handshake |
| 5 | int | Female | Right | highfive |
| 6 | int | Female | Right | request |
| 4 | int | Female | Left | handshake |
| 5 | int | Female | Left | highfive |
| 6 | int | Female | Left | request |
| 7 | com | Male | Right | Ok |
| 8 | com | Male | Right | peace |
| 9 | com | Male | Right | thumbsup |
| 7 | com | Male | Left | Ok |
| 8 | com | Male | Left | peace |
| 9 | com | Male | Left | thumbsup |
| 10 | com | Female | Right | Ok |
| 11 | com | Female | Right | peace |
| 12 | com | Female | Right | thumbsup |
| 10 | com | Female | Left | Ok |
| 11 | com | Female | Left | peace |
| 12 | com | Female | Left | thumbsup |
