# Supplementary material for: The engaging nature of interactive gestures
Source: PLoS One. 2020 Apr 23;15(4):e0232128. doi: 10.1371/journal.pone.0232128 (PMC7179864; doi:10.1371/journal.pone.0232128)
Supplement: S1 File — (DOCX) [file pone.0232128.s007.docx]

**The engaging nature of interactive gestures**

Arianna Curioni^a^, Gunther Knoblich^a^, Natalie Sebanz^a^, Lucia Maria Sacheli^b^

^a^ Department of Cognitive Science

Central European University

Október 6. Street, 7

Budapest, 1051, Hungary

^b^ Department of Psychology and Milan Center for Neuroscience (NeuroMi)

University of Milano-Bicocca

Piazza dell'Ateneo Nuovo 1

Milano, 20126, Italy.

**Corresponding author:**

Arianna Curioni

Department of Cognitive Science

Central European University

Oktober 6. Street, 7

Budapest, 1051, Hungary

[curionia@ceu.edu](mailto:curionia@ceu.edu)

**Supplementary Materials**

**Validation of the experimental stimuli: go/no-go task**

In order to ensure that the stimuli set (Interactive and Communicative) employed in our series of experiments did not differ in terms of their salience and valence for the observer, we ran a control task where we measured the time and accuracy in detecting each gesture when randomly paired with all other gestures of the stimuli set. This perceptual task provides an independent behavioural measure of the difficulty of stimulus detection. All gestures are matched for salience and valence, detection performance (accuracy and response times) across gesture categories and across individual gestures should be very similar.

**Methods**

**Participants**

Fourteen participants were recruited to take part in the experiment (12 f, average age = 23.6 years, SD age = 3.3 years). All participants reported to be right-handed and to have normal or corrected-to-normal vision. They signed prior informed consent and received monetary compensation. The study was performed in accordance with the Declaration of Helsinki and later amendments.

**Stimuli and Apparatus**

The same set of stimuli employed in the experiments were used. It comprised full-colour pictures of interactive and communicative gestures. For each condition (Interactive and Communicative) there were three different gestures (see Figure 1a), performed by a male and female model (for a total of 12 stimuli: 3 communicative and 3 interactive gestures performed by a female or male actor). The body stimuli were 500 x 296 pixels, 2.5 cm in height and 4.2 cm in width and subtended 2.6° and 4.37° of visual angle at a viewing distance of 55 cm.

The experimental script was run and participants’ responses were recorded using MatLab 16b software running on a Dell Precision T5610 PC with a screen size of 24 inch and display resolution of 1920 x 1080 at 60Hz.

**Procedure**

Task instructions were presented on the computer screen, followed by a practice trial. Participants were instructed to pay attention to a stimulus-target (gesture) presented at the beginning of each experimental block and try to remember it. Their task was to press the space bar as fast as possible when they saw the target stimulus, and refrain from responding when any other picture was presented on the screen.

At the beginning of each block a different target image was presented, and participants were given time to look at it as long as they need it before starting the experiment. After the participant pressed any key the trial started. Each trial started with a fixation cross presented at the center of the screen for 1 s. The stimulus was then presented in the center of the screen and displayed until participants had responded, for a maximum of 1s; if a response was not detected within 1 s the script proceeded to the next trial. After an inter-trial interval (ITI) that varied randomly between 1500-1900 ms after the response, the next trial started. Response Times and Accuracy of response was recorded at each trial.

**Experimental Design**

Participants completed 12 experimental blocks of 72 trials each for a total of 864 trials. In each block all 12 stimuli were randomly presented 6 times. In each block, only one stimulus was the target participant had to respond to. Trial order was randomized within and across blocks. The order of blocks was randomized across participants. Each experiment lasted approximately 20 minutes.

**Results**

The Accuracy and Response Times in detecting each gesture (in the block where it was the target) are summarized in Table S1. The repeated measures ANOVA on Reaction Times with Stimulus number (12) as within- subject factor showed no significant main effect (F(1,11) = 0.55, *p* = .864).

We also compared the mean Accuracy (Acc) and Reaction Times (RTs) between Stimulus Category (Interactive, mean Acc 0.88 +/- 0.12, mean RTs 645.32 +/- 85.37; Communicative, mean Acc 0.91 +/- 0.10, mean RTs 639.58 +/- 90.30). The non-parametric Wilcoxon test comparing mean Acc between Stimulus Categories showed no significant effect (W = 41.5, *p* = 0.17). The paired-sample t-test comparing mean RTs also showed no significant effect (t(13) = -.57, *p* = .58).

Finally, we performed a Bayesian Paired-Sample T-Test on RTs data to explore whether the data provided evidence in favour of the null hypothesis (i.e., absence of difference between Stimulus categories). The results showed a Bayesian Factor (BF10) equal to 0.31, indicating a moderate evidence in favour of the null hypothesis, i.e., that the mean RTs showed by the participants at the control task were equal for Communicative and Interactive gestures, suggesting there was no difference in salience between the Stimulus categories. With regard to Acc data, the results showed only anecdotal evidence in favour of the null hypothesis (BF10 equal to 0.655).
